# Hematuria during the right heart catheterization procedure: Renal perforation as a very rare complication

**DOI:** 10.1002/ccr3.7014

**Published:** 2023-02-24

**Authors:** Yaser Jenab, Saeed Tofighi, Hossein Navid, Homan Riazi, Sahar Samimi

**Affiliations:** ^1^ Tehran Heart Center Tehran University of Medical Sciences Tehran Iran; ^2^ Academic Educational Hospital of Duisburg‐Essen University Duisburg Germany

**Keywords:** cardiac catheterization, hematuria, iliac vein

## Abstract

The occurrence of hematuria during a right heart catheterization can be a sign of renal perforation, a rare but life‐threatening complication that could be developed due to the misdirection of wire into the abdominopelvic venous plexus. We showed this complication could be managed with venoplasty of the common iliac vein.

## Graphical abstract

Renal perforation is a rare complication during right heart catheterization which is presented with hematuria and back pain. The operator must be careful during wire manipulation, and avoid pushing it if any resistance is felt to avoid complications.

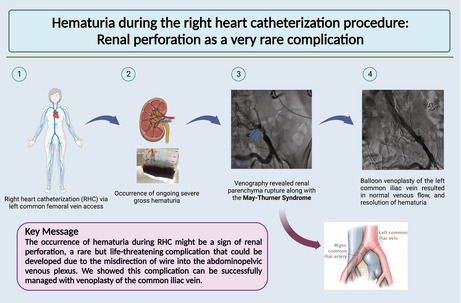

## 1 INTRODUCTION

Here we introduce a lady, undergoing right heart catheterization for her perioperative evaluations through the left femoral vein, and at the beginning of the procedure, the wire crossed from the left external iliac vein into the collateral veins and some upward branches. Afterward, the operator redirected the wire into the common iliac vein and inferior vena cava and performed the right heart catheterization. Thereafter the patient developed severe ongoing gross hematuria. Careful reevaluation of the procedure revealed a nearly occluded left common iliac vein with many collaterals indicating the unknown diagnosis of May‐Thurner syndrome in a patient with no known history of deep vein thrombosis.

Right heart catheterization (RHC) is a procedure that is commonly performed for a variety of indications, including pulmonary artery hypertension assessment and management, evaluation of valvular heart diseases, and providing a diagnostic tool for the determination of intracardiac pressures and hemodynamics, which can guide the treatment strategies for cardiologists handling heart failure patients.^1^ RHC is usually done under fluoroscopy guidance and the venous access can be via the internal jugular vein, subclavian vein, antecubital vein, or femoral vein.^2^ After vascular sheath insertion, a diagnostic angiographic catheter (often an MPA1 catheter) is introduced over a 0.035‐inch J guidewire through the common iliac vein, inferior vena cava, and then to the right atrium, right ventricle, and pulmonary artery respectively. In a study by Hoeper et al.,^3^ among 7218 RHCs, the overall number of serious complications related to the procedure was 76 cases (1.1%, 95% CI = 0.8% to 1.3%), which the most frequent event being access‐site complications (hematoma and pneumothorax), arrhythmias and vasovagal reactions, and three patients (0.052%) died.

Renal perforation during heart catheterization is an extremely rare complication. Peters et al.^4^ reported a 55 years old male, who suspected dilated cardiomyopathy and was scheduled for a left heart catheterization via right femoral artery access. After the procedure, the patient developed hematuria with back pain and the multidetector CT scan revealed a retroperitoneal hematoma and renal perforation. However, to the best of our knowledge, this adverse event has not been reported during right heart catheterization to date.

Iliac venoplasty is an effective treatment for individuals with symptomatic stenosis of common iliac veins (CIV). This condition could be developed as a consequence of compression of the left common iliac vein (CIV) between the right common iliac artery (crossing anterior to the vein) and vertebral body, which is recognized as the May‐Thurner syndrome. Due to the chronic obstruction, many collateral veins including engorged cross‐pelvic and other complex vascular networks could be found in venography, which is the gold‐standard diagnostic method for establishing the diagnosis.^5^

## 2 CASE PRESENTATION

**FIGURE 1:**
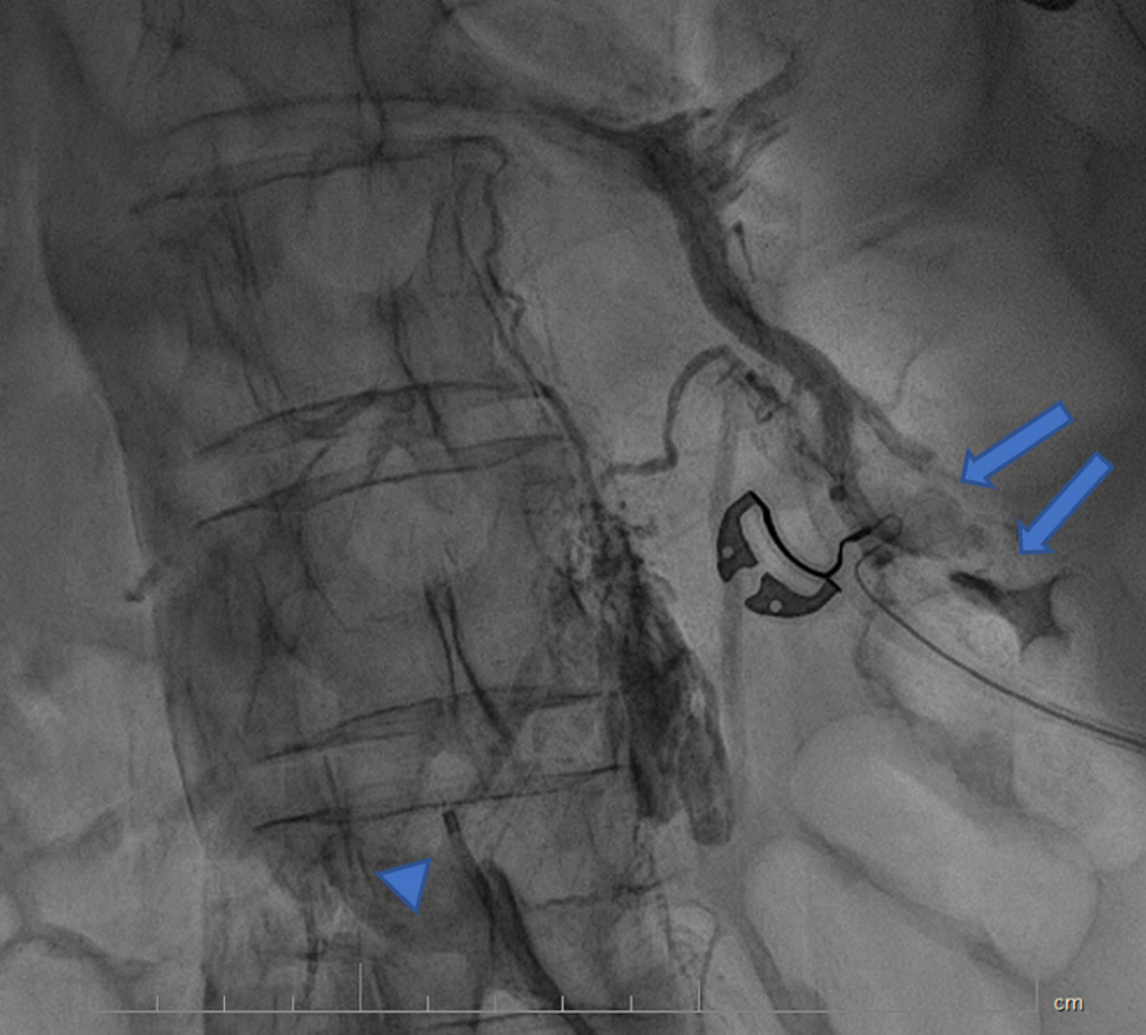
The first injection through the catheter (arrowhead) shows collateral vessels, retroperitoneal contrast accumulation, and also the entrance of contrast into the left renal pelvis (arrows).

**FIGURE 2:**
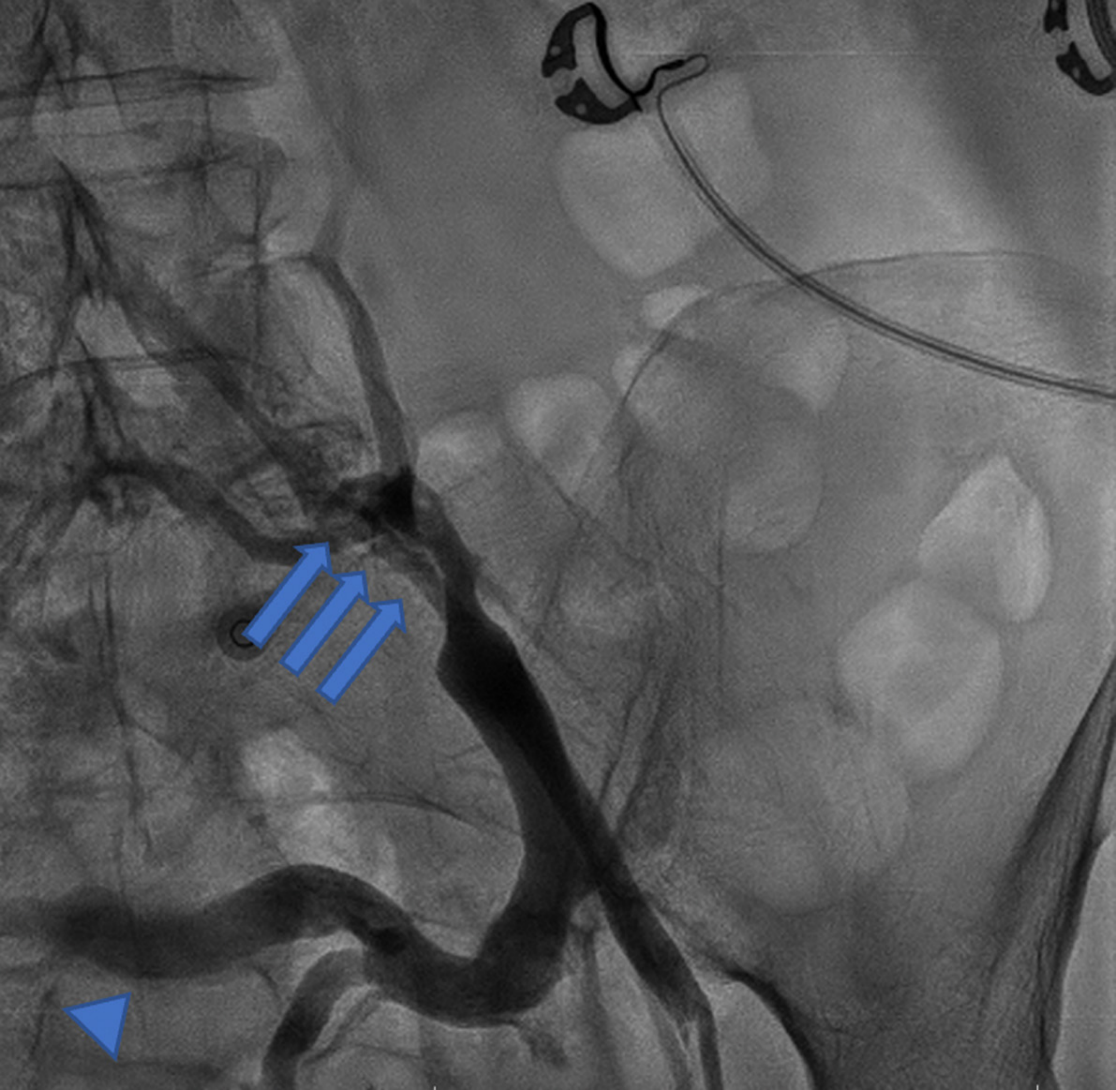
This figure illustrates severe stenosis (arrows) of the left common iliac vein with an enlarged trans‐pelvic collateral vein (arrowhead) indicating the May‐Thurner syndrome diagnosis.

**FIGURE 3:**
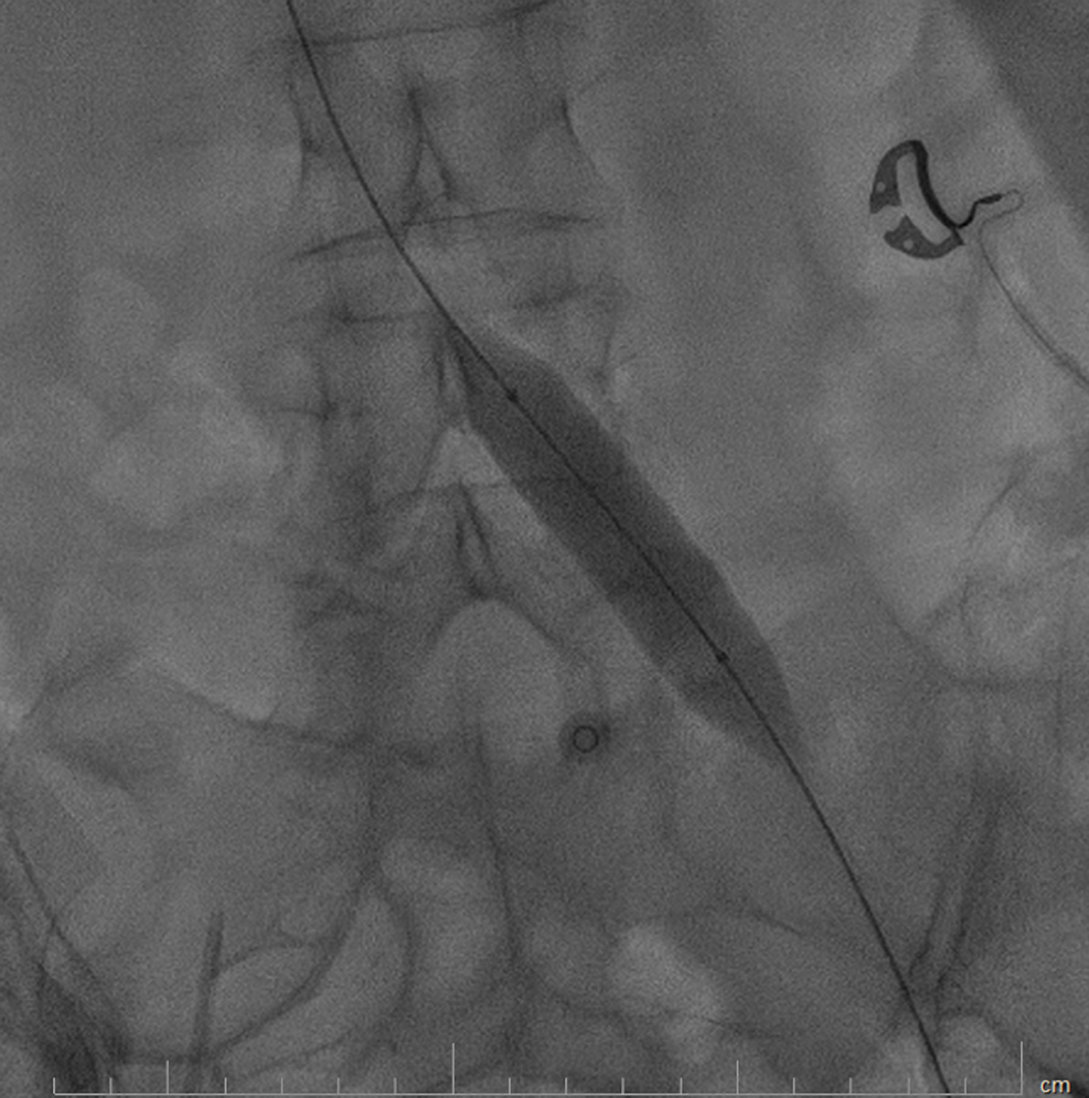
Balloon angioplasty of the left common iliac vein.

**FIGURE 4:**
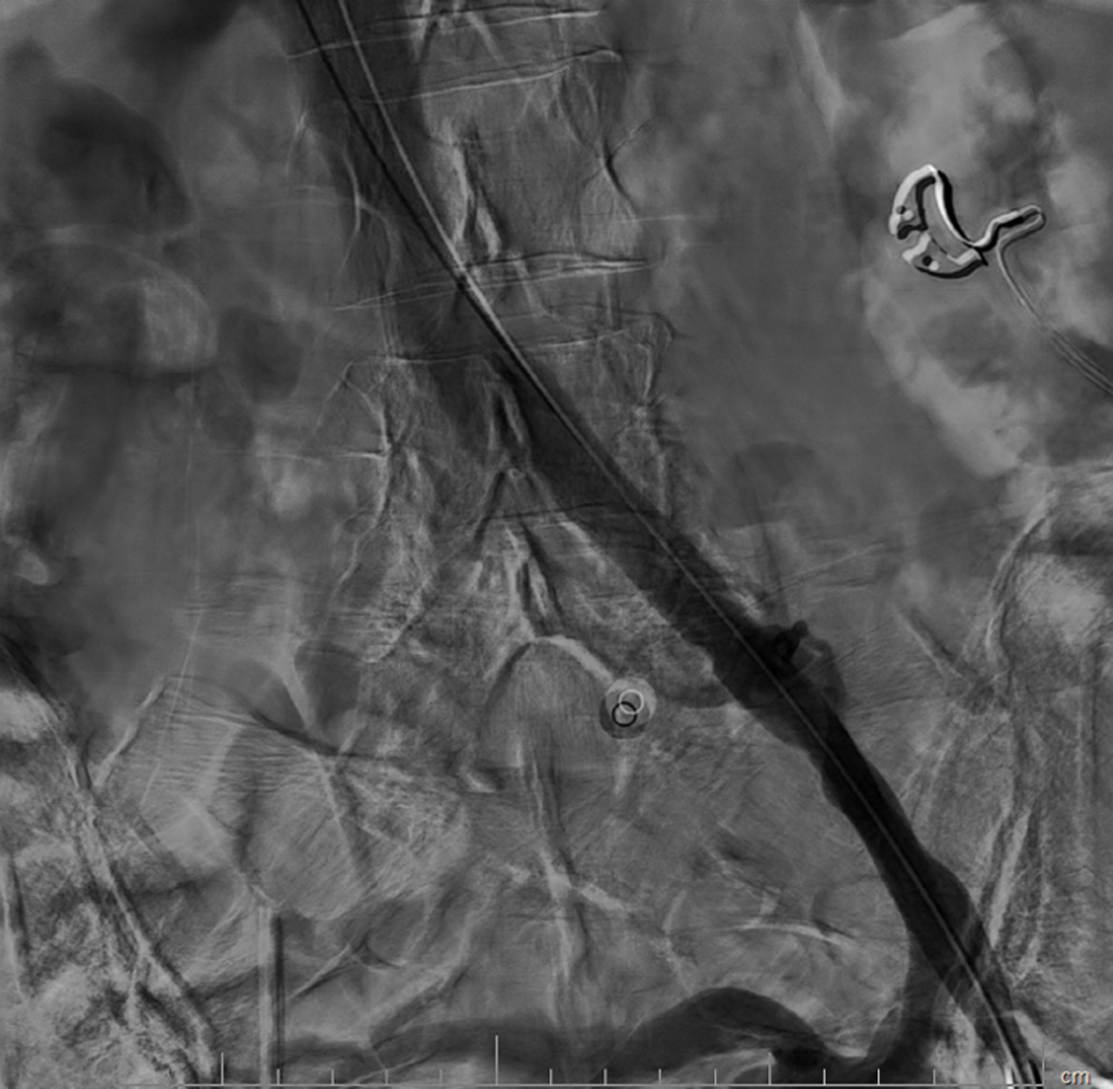
The appropriate result after balloon angioplasty of the common iliac vein with complete restoration of venous flow.

**FIGURE 5:**
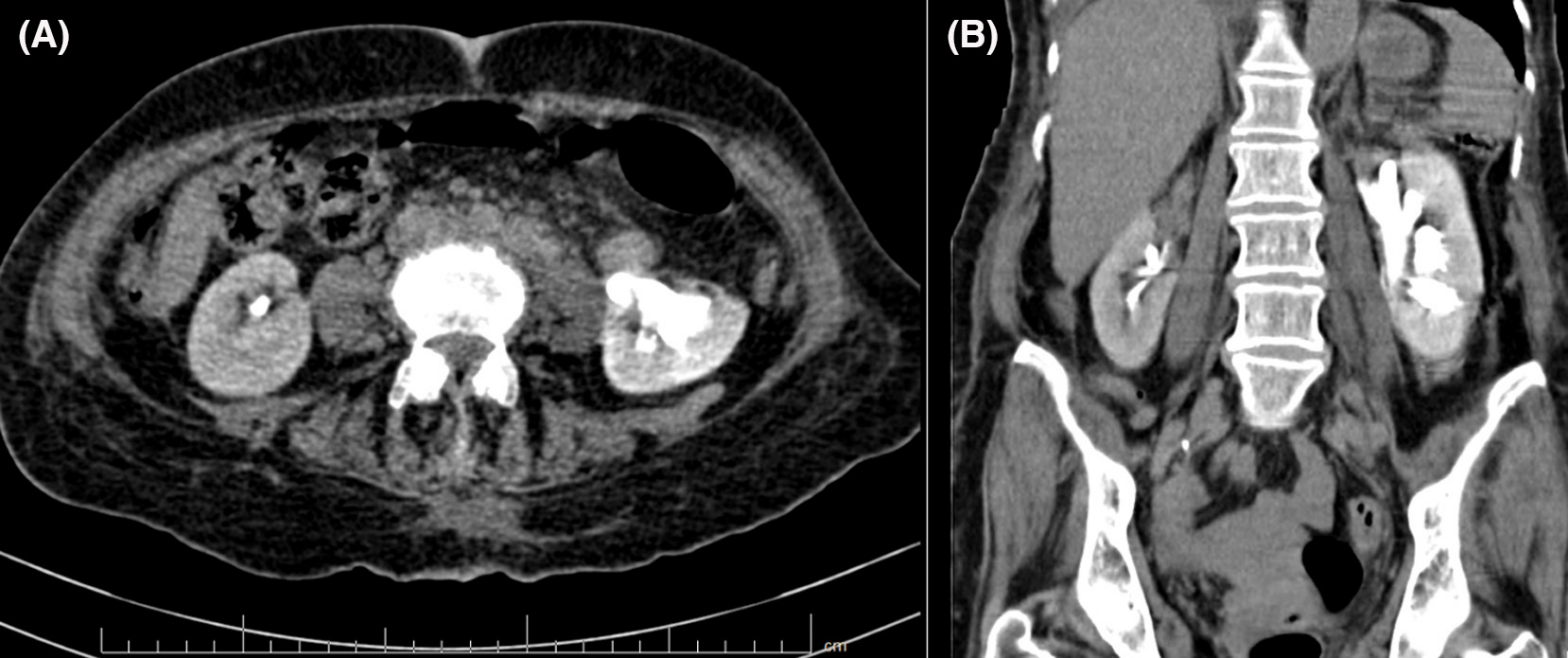
The abdominopelvic CT scan, a few hours after venoplasty, shows a significant accumulation of contrast in the left renal pelvis (compared to the normal excretion of contrast in the right kidney), without any signs of ongoing retroperitoneal hemorrhage or perinephric collection (A: Axial view, B: Coronal view).

A 70 years old lady with a history of breast cancer and mastectomy (7 years ago), who was diagnosed with severe aortic valve stenosis, became a candidate for aortic valve replacement (AVR) surgery and was scheduled for perioperative assessments including a right heart catheterization. The procedure started with a left common femoral vein (CFV) access because the right CFV had a central venous line (CV line) in place. After advancing the 0.035‐inch J wire and some wire manipulation, resistance to the crossing was felt by the operator, so an injection was done using a right Judkins catheter (Figure 1). The operator continued wire manipulation to cross left CIV and IVC. The right heart catheterization was done successfully, however, the patient had gross hematuria after the procedure. Careful re‐evaluation of images showed the contrast entrance into the left renal major calyces, through collateral vessels of the left common iliac vein severe stenosis of the left common iliac vein (LCIV), which was consistence with the May‐Thurner syndrome (MTS). The patient's hemodynamics was stable, with a blood pressure of 135/85 mmHg, a heart rate of 78 beats/min, and arterial oxygen saturation of 96%. Regarding the diagnosis of MTS, and continuation of severe gross hematuria, the heart team decided to perform balloon venoplasty of the left common iliac vein with peripheral intervention balloons including the Cronus 6*40 mm (Rontis Medical) and the AltoSa XL 16*50 mm (AndraTec) (Figures 2 and 3). Results after the procedure showed an appropriate opening of LCIV, with no remaining stenosis, and the mentioned collateral which led to blood extravasation disappeared (Figure 4). The venoplasty procedure was successful with good final results, but the stenting of LCIV wasn't performed since the patient had ongoing gross hematuria and we could not administer full anticoagulation. Regarding the results, the patient then closely followed up, and fortunately, after 3 h, the hematuria stopped. The patient was hemodynamically stable, free of pain, and serial hemoglobin measurement did not show any drop. Abdominopelvic CT scan without contrast demonstrated significant contrast accumulation in the left renal pelvis and calyces compared to normal contrast excretion on the right side. Also, there were not any signs of extravasation of contrast, or perirenal collection suggestive of ongoing bleeding (Figure 5). Three days later, the patient was discharged from the hospital in good general condition.

## 3 DISCUSSION

May‐Thurner syndrome (MTS) is a condition in which the left common iliac vein is compressed between the right common iliac artery and the lumbar vertebrae. The chronic pulsatile pressure on the vein leads to endothelial damage, intimal proliferation, and fibrin replacement, resulting in luminal stenosis and fibrosis, and in severe cases, complete occlusion would be expected.^6^ The prevalence of this anatomical variation is reported as up to 20% in the general population,^7^ and over 25% of the asymptomatic population show more than 50% stenosis of LCIV in their screening CT‐scan.^8^ The symptoms could include a range from asymptomatic iliac vein stenosis, to symptoms of chronic vein stasis of the lower limb such as pain, swelling, varicose veins, and pelvic congestion syndrome. It's also possible to develop acute deep vein thrombosis (DVT), with the following complications such as post‐thrombotic syndrome.^9^ MTS often occurs in women in their 20 and 30s, and is more common in the left leg, although some cases of the right side or bilateral MTS have been reported.^10^ The diagnosis of MTS is based on initial duplex ultrasound for assessment of the presence of any DVT, but the optimal visualization of the venous system is made by CT or MR venography, which let us directly visualize the compressive effect of the artery on the vein, and also the possible collateral formation due to chronic obstruction of the venous return flow.^11^ However, the gold‐standard test for diagnosis is invasive venography, especially with IVUS usage, where we can preciously determine the intraluminal area and diameters.^12^ Most of the patients initially have lower limb venous hypertension but have not any symptoms, but gradually they would experience some tightness and heaviness in their legs, especially after long hours of standing. The recommended treatment for MTS is venoplasty with a stent implantation.^13^ In a systematic review of 1569 patients with an established diagnosis of MTS, the endovascular interventions were performed more than surgical procedures or medical treatment, while the results showed open surgery was associated with significantly higher complications compared to interventional procedures (*p* = 0.021).^14^ In a study by Khalid Bashar et al.,^15^ 5 years follow‐up of 782 patients after stenting of the CIV due to symptomatic non‐thrombotic iliac vein lesions, showed a 94% of stent patency rate.

We faced a previously unknown case of MTS, with several pelvic collaterals, which led to the misdirection of the wire into the renal parenchyma. Jenab et al.^16^ reported a 40 years old lady with a previously known history of symptomatic MTS, who had undergone iliac venoplasty with stenting, and during the procedure, a secondary spinal epidural hematoma was developed because of the misdirection of a 0.035‐inch hydrophilic stiff wire into the paraspinal space throughout the collateral network of vertebral‐pelvic venous plexus. The patient complained of severe back pain 12 hours after the procedure and developed an abrupt loss of sensory‐motor function in the upper and lower extremities. MRI showed the epidural hematoma from cervical to lumbar spaces, which was treated with emergent laminectomy and hematoma removal. In our case, as a result of the inappropriate wiring, severe hematuria was developed, due to continuous blood flow, from a higher‐pressure space of the iliac vein into a lower‐pressure space of the renal pelvis. Lattimer et al.^17^ studied the hemodynamics of the femoral vein in individuals with venous outflow resistance (such as MTS patients) and concluded that in these circumstances, hemodynamic velocity parameters of the femoral vein are markedly increased. With this regard, the heart team decided to perform balloon venoplasty of the left CIV. After the procedure, the collateral vessels were gone, possibly due to a significant decrease in the femoral vein pressure. A few hours later, hematuria was stopped as a result of the cessation of collateral blood flow.

## 4 CONCLUSION

Femoral vein access for interventional procedures can be associated with serious complications in patients with May‐Thurner syndrome regarding their complex abdominopelvic collateral vessels. The correct direction of the wire must be achieved with precision. It's crucial that the operator manipulates the wire carefully and does not push it forward if there is any resistance to the advancement, to avoid such complications. Back pain with concomitant hematuria during the procedure can be a result of renal parenchymal perforation, which is life‐threatening and mandates emergent treatment. We illustrated this rare complication can be treated with iliac venoplasty, nevertheless, open surgery should be considered in the case of resistant ongoing hematuria.

## AUTHOR CONTRIBUTIONS

**yaser jenab:** Project administration; supervision. **Saeed Tofighi:** Investigation; writing – original draft; writing – review and editing. **Hossein Navid:** Resources. **Homan Riazi:** Supervision. **Sahar Samimi:** Writing – review and editing.

## FUNDING INFORMATION

The present report received no funding support.

## CONFLICT OF INTEREST STATEMENT

The authors have no conflict of interest to declare.

## ETHICS STATEMENT

The protocol of this study is in line with the 2013 Helsinki declaration and was approved by the Ethics Committee of Tehran University of Medical Sciences. Informed consent was taken from the patient.

## CONSENT

Written informed consent was obtained from the patient to publish this report in accordance with the journal's patient consent policy.

## Data Availability

The data that support the findings of this study are available from the corresponding author upon reasonable request.
